# Vertebrate biodiversity via eDNA at the air-water interface

**DOI:** 10.1016/j.isci.2026.115682

**Published:** 2026-04-09

**Authors:** Yin Cheong Aden Ip, Pedro F.P. Brandão-Dias, Gledis Guri, Elizabeth Andruszkiewicz Allan, Ryan P. Kelly

**Affiliations:** 1 School of Marine and Environmental Affairs, University of Washington, Seattle, WA, USA

**Keywords:** Environmental science, Biological sciences, Zoology, Nucleic acids

## Abstract

Aquatic, aerial, and terrestrial habitats exist along a continuum, with biomass and energy flows transporting genetic material across environmental boundaries. Here, we use environmental DNA (eDNA) metabarcoding to characterize genetic information exchange between water and air. From 27 paired samples collected at two urban-wildland interface sites using passive air sampling and active water filtering, we recovered 35 vertebrate taxa, with 40% detected in both media, ranging from aquatic salmon to terrestrial cottontail rabbit. Cross-medium detection probability scales with DNA abundance: logistic models identify ∼660 water reads and ∼14 air reads as 50% detection thresholds. Peaks in coho and Chinook salmon eDNA in water and air align within 24 h, demonstrating that passive air sampling reflects temporal abundance trends. Low-abundance taxa appear sporadically, reflecting stochastic behavior at low DNA concentrations, and reliable detection requires intensified sampling in the primary habitat. Together, these findings establish a unified framework for holistic vertebrate biodiversity monitoring at the land-water interface, with applications in conservation, invasive species early warning, and One Health surveillance.

## Highlights

• Paired air-water eDNA metabarcoding detects 35 vertebrate taxa
• 40% of taxa detected in both air and water sampling media
• Cross-medium detection follows abundance-dependent thresholds
• Salmon eDNA peaks synchronize across water and air within 24 h

## Graphical abstract

**Figure:**
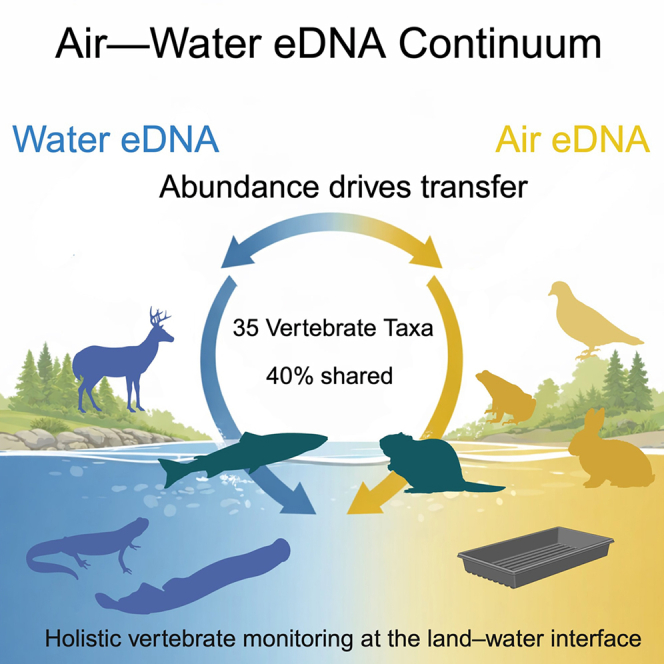

## Introduction

Multicellular species continually shed genetic traces into their surroundings, leaving environmental DNA (eDNA) in water and air. For the most part, eDNA surveys treat these two reservoirs as separate, as researchers assume that water samples predominantly yield aquatic species, and air samples predominantly yield terrestrial taxa. For instance, water samples routinely reveal fish, amphibians, and macroinvertebrates,^1^^,^^2^ and air samples recover bird, mammal, and plant DNA from the atmosphere.^3^^,^^4^ As a result, “exogenous” signals—DNA from organisms not resident in the sampled medium—are often dismissed as incidental or ignored.

In reality, physical and biological processes routinely transport genetic material across environmental boundaries through a variety of mechanisms. Rainfall and overland flow can carry terrestrial DNA into streams; surface splashes and bubble-burst aerosolization propel waterborne DNA skyward; windblown dust and settling particles shuttle DNA molecules among air and water.^5^^,^^6^ Consequently, trace signatures of nonresident taxa are expected to appear in eDNA surveys. In this context, we define “exogenous eDNA” as genetic material detected in a medium where the source organism is not resident—for instance, terrestrial mammals in water samples and aquatic fish DNA in air filters.^7^^,^^8^^,^^9^ Such exogenous detections can arise not only from abiotic transport but also from biotic and human vectors.^10^ Predators and scavengers may carry prey DNA across habitats, and human activities such as bait fish disposal, livestock grazing near banks, or urban runoff of food waste can introduce non-resident DNA into aquatic and airborne environmental samples.^11^ Because eDNA studies typically target species native to the sampled medium, such cross-compartment signals are difficult to interpret without additional ecological context. Incorporating background ecological knowledge and land-use history is therefore essential to distinguish genuine continuum signals from vector-driven deposits and to interpret unexpected detections responsibly.

Our recent quantitative analysis of cross-medium genetic signal transfer showed that spawning salmon release detectable aquatic eDNA into the atmosphere and that simple passive air samplers recover fish-derived DNA sequences related to visual fish counts.^7^ Meanwhile, multiple prior airborne eDNA studies in purely terrestrial contexts have demonstrated that passive and pump-assisted air collectors reliably recover resident species’ DNA from mammals, birds, and plants carried on ambient aerosols.^4^^,^^12^^,^^13^^,^^14^^,^^15^ These complementary lines of work highlight that airborne eDNA can originate from two distinct pathways: direct shedding by land-based and aerial species and cross-medium transfer from aquatic species. This observation points to two orthogonal axes that may structure eDNA detection: sampling medium (air vs. water) and habitat affinity (aquatic, amphibious, terrestrial). Taken together, cross-medium detections are not random but instead reflect an underlying, abundance-dependent process.

We therefore hypothesize that eDNA persists in pools within each environmental partition (water or air) and that the probability of transfer from one partition to another is influenced by the concentration of a species’ DNA in the source partition. DNA molecules at greater concentration are more likely to move across partitions, with transfer occurring through distinct physical mechanisms depending on direction (aerosolization for water-to-air, deposition for air-to-water). Under this mechanistic model, cross-medium detections should follow predictable patterns: (1) continuum authenticity: do detections of aquatic species in air (and terrestrial species in water) reflect genuine aerosolization and deposition rather than methodological contamination? (2) abundance dependency: where a species’ DNA is more abundant in its native pool, is it more likely to appear in the alternate pool? (3) temporal synchrony: if water and air truly form a continuum of eDNA signals, will dominant taxa exhibit synchronous temporal trends across both reservoirs? (4) sampling effort: because rare species shed little DNA, how much sampling effort is required to reliably recover faint signals in both media?

To address these questions, we conducted the first side-by-side metabarcoding survey of vertebrate eDNA at the air-water interface. Over seven consecutive weeks, we deployed paired open-water trays for passive air sampling and active river-grab filters for water sampling at two urban-wildland interface sites. By comparing taxon co-occurrence, modeling cross-medium detection probabilities, and examining time-series alignment, we evaluate whether water and air truly function as a unified genetic reservoir. This framework lays the groundwork for a unified, noninvasive approach to survey vertebrate life at the land-water interface, with applications in conservation, invasive species early warning, and One Health surveillance.

## Results

### An eDNA continuum

**Figure 1:**
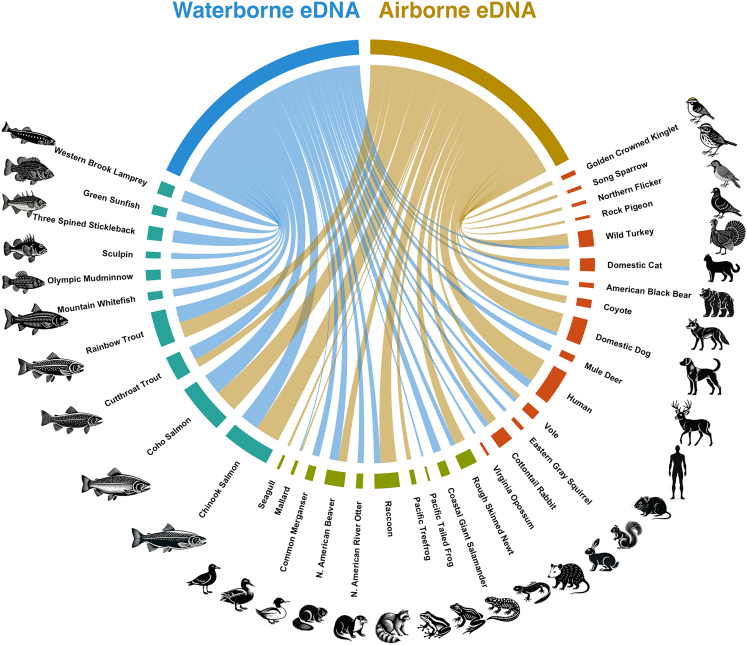
Chord diagram showing overall cross-medium eDNA signal between water and air Chord ribbons connect the two sampling media—blue arc = water eDNA; gold arc = air eDNA—linked to each detected taxon, with ribbon thickness proportional to the log_10_-transformed total read count recovered. Outer arc segments are colored by habitat affinity: teal = aquatic, olive = amphibious, and earth-tone = terrestrial.

We conducted 17 sampling trips over 7 weeks, yielding 27 eDNA collections. These comprise 14 airborne (open-water trays) and 13 waterborne (river-grab filters) samples, which together generated 254, 252 high-confidence reads after operational taxonomic unit (OTU) curation (Table S1). Across all samples, we detected 35 vertebrate taxa after excluding four putative “food” items: northern anchovy (*Engraulis mordax*), Pacific sardine (*Sardinops sagax*), European sprat (*Sprattus sprattus*), and wild boar/domestic pig (*Sus scrofa*) (Table 1). The three clupeids are marine species and cannot occur at our freshwater sites, and *Sus scrofa* is not established locally; these reads most plausibly reflect human food or bait inputs rather than local fauna. Of the 35 taxa, 14 species (40%) were detected in both water and air (e.g., coho salmon [*Oncorhynchus kisutch*], mallard [*Anas platyrhynchos*], North American beaver [*Castor canadensis*], domestic dog [*Canis lupus familiaris*]); 14 (40%) appeared only in water (e.g., western brook lamprey [*Lampetra richardsoni*], three-spined stickleback [*Gasterosteus aculeatus*]); and 7 (20%) were detected only in air (e.g., Pacific treefrog [*Pseudacris regilla*], song sparrow [*Melospiza melodia*]) (Figure 1, Table 1). The chord diagram illustrates a continuum: blue (water) and gold (air) ribbons interweave into a dense web of shared taxa, while the outer arcs highlight each medium’s unique signals and species’ habitat types. Ribbon widths are scaled to log_10_(reads +1) and are dominated by a handful of abundant aquatic species (notably the salmon *Oncorhynchus* spp.), which together contribute a substantial fraction of the total eDNA flux in both air and water. Using only presence/absence data, a Pearson’s chi-square test on a 3 × 2 table of habitat affinity (aquatic, amphibious, terrestrial) versus detection medium (air vs. water) found no significant association (χ^2^ = 1.57, df = 2, *p* = 0.46), indicating that, when treating each species as simply “present” or “absent,” the proportion detected in air versus water does not differ by habitat affinity.

**Table 1:** Vertebrate taxa sorted by media in which they were detected (excluding four “food” species)

Detection media	Habitat affinity/Biology	Common Name	Species
Shared	Amphibious	Mallard	*Anas platyrhynchos*
Shared	Terrestrial	Domestic dog	*Canis lupus*
Shared	Amphibious	North American Beaver	*Castor canadensis*
Shared	Terrestrial	Domestic Cat	*Felis catus*
Shared	Terrestrial	Human	*Homo sapiens*
Shared	Terrestrial	Wild Turkey	*Meleagris gallopavo*
Shared	Terrestrial	Vole	*Microtus* spp.
Shared	Terrestrial	Cottontail Rabbit	*Sylvilagus* spp.
Shared	Amphibious	Rough-skinned Newt	*Taricha granulosa*
Shared	Aquatic	Cutthroat Trout	*Oncorhynchus clarkii*
Shared	Aquatic	Coho Salmon	*Oncorhynchus kisutch*
Shared	Aquatic	Rainbow Trout	*Oncorhynchus mykiss*
Shared	Aquatic	Chinook Salmon	*Oncorhynchus tshawytscha*
Shared	Amphibious	Raccoon	*Procyon lotor*
Water-only	Amphibious	Coastal (Pacific) Tailed Frog	*Ascaphus truei*
Water-only	Aquatic	Sculpin	*Cottus* spp.
Water-only	Amphibious	Coastal Giant Salamander	*Dicamptodon tenebrosus*
Water-only	Terrestrial	Virginia Opossum	*Didelphis virginiana*
Water-only	Aquatic	Three-spined Stickleback	*Gasterosteus aculeatus*
Water-only	Aquatic	Western Brook Lamprey	*Lampetra richardsoni*
Water-only	Aquatic	Green Sunfish	*Lepomis cyanellus*
Water-only	Amphibious	North American River Otter	*Lontra canadensis*
Water-only	Amphibious	Common Merganser	*Mergus merganser*
Water-only	Aquatic	Olympic Mudminnow	*Novumbra hubbsi*
Water-only	Terrestrial	Mule Deer	*Odocoileus hemionus*
Water-only	Terrestrial	Eastern Gray Squirrel	*Sciurus carolinensis*
Water-only	Aquatic	Mountain Whitefish	*Prosopium williamsoni*
Water-only	Terrestrial	American Black Bear	*Ursus americanus*
Air-only	Terrestrial	Coyote	*Canis latrans*
Air-only	Terrestrial	Northern Flicker	*Colaptes auratus*
Air-only	Terrestrial	Rock Pigeon	*Columba livia*
Air-only	Amphibious	Seagull	*Larus* spp.
Air-only	Terrestrial	Song Sparrow	*Melospiza melodia*
Air-only	Amphibious	Pacific Treefrog	*Pseudacris regilla*
Air-only	Terrestrial	Golden-crowned Kinglet	*Regulus satrapa*

The read counts for these taxa are available in Table S2 and Data S2. All no-template controls (NTCs) sequenced across PCR runs produced low read counts dominated by bacteria, with scattered geographically implausible vertebrate assignments (Data S3). No salmonid reads were detected in any NTC. Field blanks yielded trace vertebrate reads (e.g., *Lampetra richardsoni*, waterfowl, and small mammals) and rare, low-copy salmonid detections (e.g., *O. kisutch*, *O. tshawytscha*), consistent with background deposition rather than laboratory contamination. The full detection list and read counts are provided in Table S2.

### Cross-medium detection thresholds

**Figure 2:**
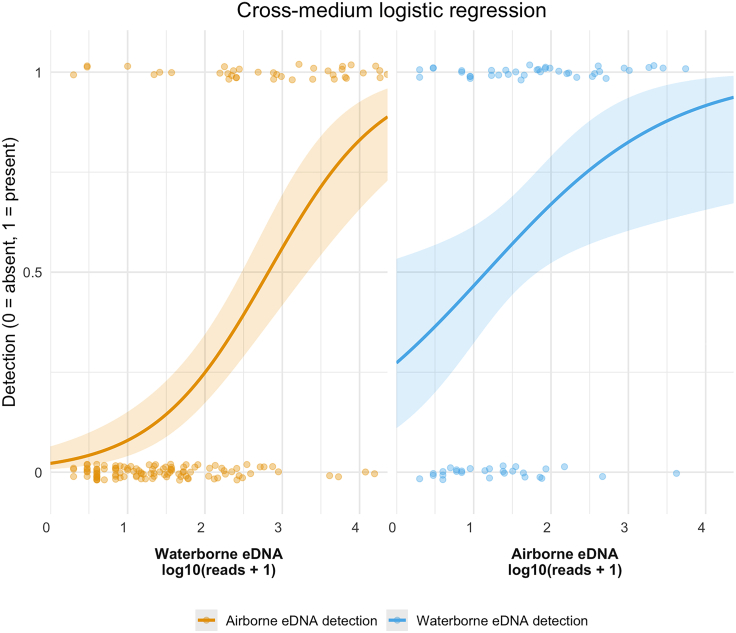
Cross-medium logistic regression of eDNA detection probability In the left panel, for each species detected in water, golden points indicate whether that species was also detected in air (1 = present, 0 = absent) at each log_10_-transformed waterborne read count. The golden curve is the fitted binomial generalized linear model (GLM), illustrating how higher water eDNA abundance predicts a greater probability of exogenous transfer and detection in air. In the right panel, blue points and curve show, for species detected in air, the probability of detecting the same species in water as a function of its airborne eDNA counts. Points are jittered vertically for clarity. Shaded ribbons around each curve represent the 95% confidence intervals of the logistic regression fit.

For waterborne-to-airborne eDNA transfers, the logistic regression model yielded an intercept β_0_ = −3.79 and slope β_1_ = 1.35 (SE = 0.235, *p* = 1.08 × 10^−8^) on the logit scale, corresponding to a 50% detection probability at log_10_(water_reads +1) ≈ 2.82 (≈660 raw reads). For airborne-to-waterborne eDNA transfers, we obtained β_0_ = −0.98 and β_1_ = 0.844 (SE = 0.339, *p* = 0.013) on the logit scale, with the 50% threshold at log_10_(air_reads +1) ≈ 1.16 (≈14raw reads) (Figure 2). Habitat-specific logistic curves with 95% confidence intervals (CIs) are provided in Figure S1.

The ∼660 water reads and ∼14 air reads values denote 50% detection probabilities for this device (MinION R10.4.1 flowcells), marker (MiFish-U 12S), and sequencing depth (∼35,800 raw reads per sample on average; Table S1). These are operational thresholds specific to our sampling configuration and are not intended as generalizable concentration cutoffs across sites, samplers, or taxa. Given the differing sampling effort (1 L water vs. 750 cm^2^ · 24 h deposition) and the absence of volumetric air-flow measurements, we summarize cross-medium patterns as co-detections and presence/absence rather than direct contrasts of read counts.

The logistic regression shows that once eDNA abundance exceeds medium-specific thresholds, cross-medium detection becomes more predictable, consistent with mechanistic aerosolization and deposition rather than sporadic artifacts. In the water-to-air regression (Figure S1), fully aquatic taxa (Coho and Chinook salmon) exhibit a steep slope: once waterborne reads exceed the threshold, the probability of detecting them in air rises sharply above 50%. Amphibious species have a much shallower slope and do not reach a 50% chance of airborne detection at any water-read level. Terrestrial taxa remain at zero transfer probability in the water-to-air model, suggesting that strictly land-based species detected in air arise from direct shedding rather than aquatic aerosolization. Conversely, the air-to-water model (Figure S1) shows that aquatic taxa are consistently detected in water, amphibious taxa rise gradually, and terrestrial taxa require high airborne counts. Together, these patterns confirm that cross-medium detection is fundamentally abundance-driven. A few low-read outliers (e.g., terrestrial DNA in water) reveal genuine “reverse” transfers and emphasize the need to interpret exogenous detections in the context of local ecology and hydrology.

### Temporal synchrony across media

**Figure 3:**
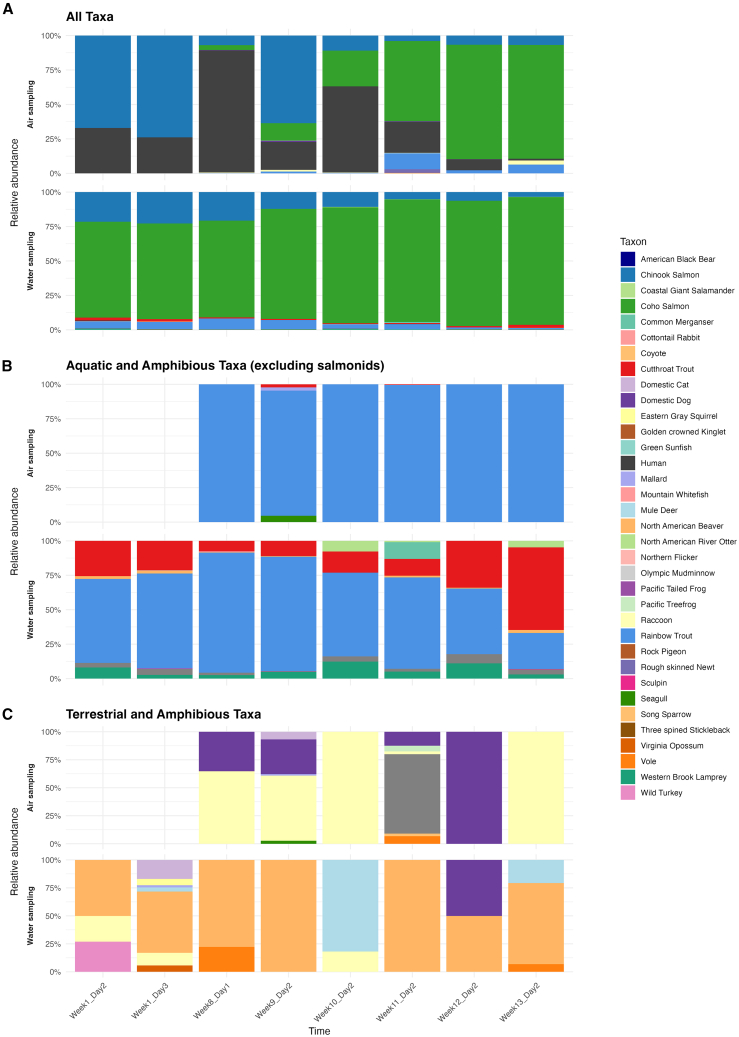
Relative abundance of eDNA-detected taxa at Issaquah Hatchery over time, comparing air versus water sampling (A) All detected taxa, illustrating salmonid dominance (Coho and Chinook salmon comprise 60%–90% of reads). (B) Aquatic and amphibious taxa with Coho and Chinook salmon and humans removed, revealing Rainbow Trout and Cutthroat Trout as the next most abundant species. (C) Only terrestrial and amphibious taxa, omitting humans. In each panel, the top row (air sampling) and bottom row (water sampling) show stacked barplots of the proportional (100%) taxon composition over 8 weeks. Taxa are keyed by color in the legend to the right.

**Figure 4:**
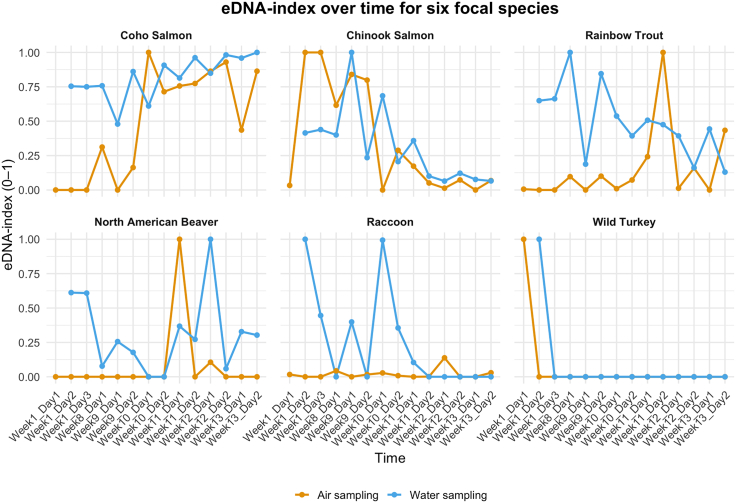
Temporal dynamics of eDNA index for six focal species Each panel shows the normalized eDNA index (0–1) across the time (week_day). Gold lines and points represent airborne eDNA sampling; blue lines and points represent waterborne eDNA sampling, illustrating whether both lines match or track one another. (A)–(F) correspond to Coho Salmon, Chinook Salmon, Rainbow Trout, North American Beaver, Raccoon, and Wild Turkey, respectively, illustrating how relative abundance in each medium varies over time. A point at y = 0 indicates a sample was collected but yielded no reads for that species on that date; absence of a point indicates no sample was taken.

Stacked-bar time-series at Issaquah Hatchery (Figure 3) and Confluence Park (Figure S2) summarize proportional community composition in air and water, showing the relative abundance of detected taxa at each sampling time point. For example, when all taxa are considered (Figure 3A), salmonids (Chinook and Coho) together comprise >70% of reads in both media, especially in weeks 8–10 during the peak spawning run. Pulses of terrestrial detections (e.g., North American Beaver, Mule Deer, and Wild Turkey) also appear in both media at roughly the same times. Focusing on aquatic and amphibious species (Figure 3B) still shows that salmonid signals rise and fall concurrently in air and water, and even terrestrial and amphibious taxa (North American Beaver and Raccoon; Figure 3C) tend to exhibit their major peaks in both media around the same dates. A few minor deviations, such as a slightly stronger Pacific Treefrog signal in air compared to water during week 11, reflect that low-abundance eDNA signals can decouple briefly between media, since rare taxon detection is inherently unpredictable. This pattern of temporal cross-medium concordance is most evident for common species. For rarer taxa, concordance becomes more stochastic due to both biological factors (sporadic presence) and mathematical factors (lower eDNA index precision). Confluence Park (Figure S2) shows a very similar pattern: overall community shifts occur in both air and water at the same general times, but individual proportions sometimes differ. We next examine per-species synchrony between media using eDNA-index trajectories (Figure 4).

### Focal-species eDNA-index reveals synchronized dynamics

Temporal concordance for six focal species is shown in Figure 4, where air and water eDNA-index trajectories are plotted together to visualize synchrony. We selected six focal taxa based on their high total read counts and consistent detection in both air and water: Coho Salmon, Chinook Salmon, Rainbow Trout, North American Beaver, Raccoon, and Wild Turkey, thereby representing aquatic, amphibious, and terrestrial habitat affinities. For each species, we computed an eDNA index^16^ and plotted its time series across all sampling dates (Figure 4).

Since water samples were collected at the start of each trip, immediately before deploying the 24 h passive air traps, any apparent 1-day offset between waterborne and airborne peaks reflects our sampling schedule rather than a true biological lag in eDNA transfer. Coho Salmon exhibited synchronized peaks during the main spawning period at week 9, with water reaching an index ≈0.95 at week 9 day 2 and air reaching an index of 1.0 at week 10 day 1. Chinook Salmon showed closely aligned peaks, with both media reaching an index of 1.0 around weeks 8–9, with air peaking at week 9 day 1–2 and water at week 9 day 1, reflecting the overlapping spawning window. Rainbow Trout exhibited decoupled dynamics, with water peaking early at week 8 day 1 (index = 1.0), followed by a secondary pulse around week 9 day 2 (index ≈0.85), while air showed a delayed maximum at week 11 day 2 (index = 1.0). North American Beaver spiked in water at week 12 day 1 (index = 1.0), with air peaking approximately 1 week earlier at week 11 day 1 (index = 1.0), following earlier low-level signals (index ≈0.6) in water during weeks 1–2. The amphibious Raccoon (which habitually washes its food at the creekside) displayed an isolated waterborne peak from weeks 9–10 (index ≈0.8–1.0), which was followed by a brief airborne detection in week 12. Wild Turkey was detected only at the very first sampling point in week 1 in both media and was absent thereafter.

Across our 24 h sampling window, the six focal species (selected for having sufficient detections in both media to enable paired temporal comparisons) show broadly concurrent dynamics between media. Per-species lag-0 (no lag) regressions of air eDNA-index on water eDNA-index yielded modest but positive concordance, with R^2^ spanning ∼0.00 (Wild Turkey, rare detection) to ∼0.33 (Coho Salmon). Not all species show identical agreement, as rainbow trout and raccoon display weaker or offset air-water concordance with medium-specific peaks (Figure 4), consistent with lower biomass and terrestrial behavior producing noisier or context-dependent detections. Concordance increased with species abundance: a cross-species regression of per-species R^2^ against log10(total reads +1) was positive and significant (β ≈ 0.10 ± 0.03, *p* = 0.02, adj. R^2^ = 0.48; Figure S3). Given the weekly cadence and occasional gaps (Table S1), these are descriptive lag-0 statistics; denser (daily or sub-daily) sampling would be needed to estimate temporal lags formally.

### Stochastic detection of rare taxa

**Figure 5:**
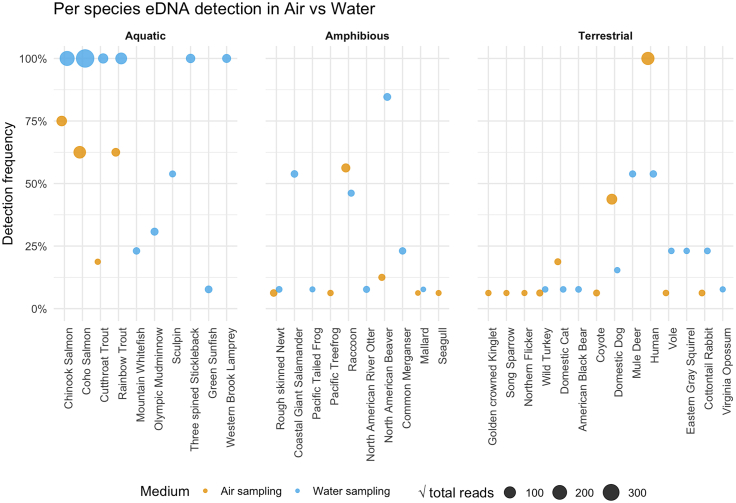
Detection frequency of eDNA for individual species in airborne vs. waterborne samples, grouped by habitat affinity Each point shows the percentage of samples in which a given species was detected (*y* axis) in air (gold) or water (blue), with point size proportional to the square root of total read count. Species are ordered and faceted by habitat affinity (aquatic, amphibious, terrestrial) to illustrate how detection success varies by medium and habitat affinity.

Detection frequency, calculated from the proportion of samples in which each taxon appears, varies strongly with total read count (scaled by √ -square-root point size; Figure 5). The most abundant species showed consistently high detection in water but more variable detection in air: coho salmon was detected in 100% of waterborne samples (13/13) and 71% of airborne samples (10/14), while Chinook Salmon appeared in 100% of water samples (13/13) and 86% of air samples (12/14). In contrast, low-abundance taxa (e.g., Mallard, Pacific Treefrog) fell below 10% detection in either medium. The tight, positive relationship between √(total reads) and detection probability implies that reliably capturing rare signals would likely require several-fold more replicates than for dominant taxa.

### Medium drives eDNA community structure

**Figure 6:**
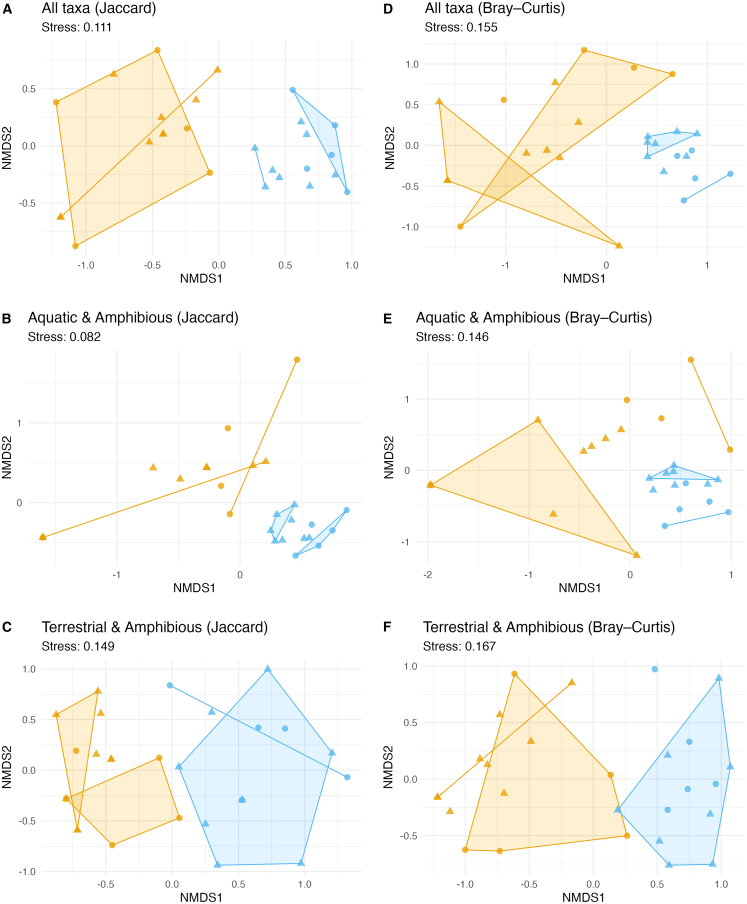
nMDS of eDNA community composition across sites and sampling media Panels show Jaccard dissimilarity (A–C) and Bray–Curtis dissimilarity (D–F). Points represent individual samples, with color indicating sampling medium (gold = air, blue = water) and shape indicating site (circle = Confluence Park, triangle = Issaquah Hatchery). Shaded polygons represent convex hulls enclosing all samples within each sampling medium. Stress values for each analysis are shown beneath the panel titles. (A) All taxa, Jaccard. (B) Aquatic and amphibious taxa, Jaccard. (C) Terrestrial and amphibious taxa, Jaccard. (D) All taxa, Bray–Curtis on log10-transformed read counts. (E) Aquatic and amphibious taxa, Bray–Curtis. (F) Terrestrial and amphibious taxa, Bray–Curtis.

Non-metric multidimensional scaling (nMDS) of both presence-absence (Jaccard; per-panel stress values 0.082–0.149) and abundance (Bray-Curtis; per-panel stress values 0.146–0.167) matrices (Figures 6A–6F) revealed that samples cluster by sampling medium (air vs. water). In the Jaccard ordination of all taxa (Figure 6A), air and water points form two distinct clouds, and the Bray-Curtis ordination (Figure 6D) shows the same pattern. Subsetting to aquatic and amphibious taxa (B, E) or terrestrial and amphibious taxa (C, F) yields similar medium-driven separation. Permutational ANOVA (adonis2, 999 permutations) on Jaccard dissimilarity (presence-absence) showed that sampling medium (air vs. water) explained the largest share of variance (R^2^ = 0.32, F = 12.8, *p* = 0.001), with site location and sampling week each contributing smaller but significant effects (R^2^ = 0.06 and 0.05, respectively; both *p* < 0.05), and day showing no effect (R^2^ = 0.02, *p* = 0.18). Analysis of Bray-Curtis dissimilarity (abundance-weighted) yielded similar results, with medium dominating variance partitioning (R^2^ = 0.44, F = 21.5, *p* = 0.001), week significant (R^2^ = 0.07, F = 2.1, *p* = 0.009), and location and day non-significant (both *p* > 0.05). These patterns confirm that medium is the primary driver of community composition, yet the substantial proportion of shared taxa (40%) and predictable cross-medium transfers indicate that air and water function as coupled compartments within a unified eDNA continuum.

## Discussion

### An air-water eDNA continuum

Our side-by-side survey of river-grab filters and passive open-water trays provides the first field-scale evidence that water and air can function as coupled reservoirs of eDNA at this site during our sampling period. While water and air samples offer partially complementary signals, aquatic and airborne eDNA co-occur for at least 40% of detected taxa, ranging from Salmon (*Oncorhynchus* spp.) to Raccoons (*Procyon lotor*), and at rates exceeding random expectation. This “genetic Ferris Wheel” likely reflects natural processes: stream turbulence and bubble-burst events likely help to aerosolize waterborne DNA; rainfall and overland flow return terrestrial signals to the river; and gravitational settling deposits airborne fragments onto surfaces.^5^^,^^6^^,^^17^^,^^18^ By revealing these linked pathways, our work breaks down the traditional water-versus-air compartments of eDNA research and supports a continuum-based perspective on genetic monitoring and ecosystems.^4^^,^^7^^,^^19^ Building on the salmon-focused qPCR work of Ip et al. (2025),^7^ which first showed that spawning salmon release detectable DNA into the air, we here broaden that insight to a fuller vertebrate community. Progressing from targeting a single species, we metabarcoded 35 taxa, and rather than sampling air alone, we paired open-water trays with river-grab filters, enabling us to estimate medium-specific, system-level detection thresholds—operational for our 1 L water grabs and 750 cm² · 24 h trays rather than species-general cutoffs. Species-specific variation in shedding, decay, and transfer efficiency is expected and may influence detection thresholds.

Water-air coupling also sits within a broader multi-modal eDNA landscape. Airborne eDNA tends to do better at detecting birds and terrestrial mammals^3^^,^^20^^,^^21^^,^^22^; water eDNA captures fish and amphibians^1^^,^^2^; and invertebrate-derived DNA (iDNA from leeches or mosquitoes) can reveal otherwise elusive species.^23^^,^^24^^,^^25^ These approaches are often treated as separate toolkits optimized for different taxa. Our results advance this view by showing that water and air are not isolated reservoirs but dynamically coupled compartments with quantifiable, abundance-driven exchange. Rather than treating cross-medium detections as artifacts to be discarded, we show they follow predictable patterns that inform sampling design and ecological interpretation. This shift from analyzing media separately to understanding a unified “genetic cloud” opens practical possibilities where a single passive air sampler can simultaneously survey terrestrial and aquatic biodiversity.

Importantly, this continuum is not random background noise but follows clear, abundance-driven rules. Because abundant DNA molecules in one compartment will always feed the next, high eDNA concentration in water inevitably produces some airborne fragments, and vice versa. Our models identify 50% detection probability thresholds of approximately 660 raw water reads to predict airborne detection, and about 14 raw air reads to predict waterborne detection. As each water sample (1 L) and each air sample (750 cm² · 24 h) represent very different sampling substrates, and we did not directly measure airborne deposition to convert into an equivalent air volume, these thresholds must be interpreted qualitatively rather than as absolute concentration comparisons. Future work should quantify deposition rates under comparable field conditions to normalize air-versus-water sampling effort and quantify species-specific eDNA mass as a predictor variable, rather than using Oxford Nanopore Technologies (ONT) read abundance as a proxy for abundance.

In the water-to-air model, fully aquatic taxa, especially those engaged in high-energy behaviors like spawning that generate bubble-burst aerosols, exhibit the steepest curves, reflecting their efficient transfer of DNA from water into air. Amphibious species, by virtue of using both aquatic and terrestrial habitats, shed DNA into each medium directly. As a result, their air-detection curves represent a combination of water-to-air transfer and direct terrestrial shedding, yielding intermediate slopes relative to fully aquatic or strictly terrestrial taxa. Truly terrestrial species (for example, Mule Deer and Wild Turkey) fall outside this water-to-air framework: their DNA never passes through the aquatic pool but is released directly into the atmosphere via hair, skin cells, or feces, producing essentially flat dose-response curves when plotted against water read counts. To capture their dynamics, we instead employ an air-to-water model in which airborne abundance predicts the occasional appearance of land-derived DNA in water through settlement, deposition, runoff, rain-splash, or overland flow.

Some vertebrate detections likely reflect anthropogenic vectors rather than natural cross-medium transport. For example, Domestic Pig (*Sus scrofa*) DNA was detected only in trace amounts and excluded from our primary analyses. The pig signal most plausibly arose from food-waste disposal, runoff, or hatchery handling. Similarly, Domestic Dog and Cat detections may reflect pet activity or food waste near sampling sites. We addressed this conservatively by (i) explicitly excluding four “food” taxa (Northern Anchovy, Pacific Sardine, European Sprat, and Domestic Pig) that cannot naturally occur in freshwater Issaquah Creek (STAR Methods, bioinformatics pipeline); (ii) retaining only detections showing co-occurrence across media, replicate consistency, and geographic plausibility; and (iii) treating human-associated species (*Homo sapiens*, domestic animals) as ambient background expected at urban-wildland interface sites. Biotic vectors such as predators and scavengers can also transport prey DNA across habitats. Distinguishing these from abiotic transport will require experimental manipulation beyond our observational scope. Incorporating land-use covariates, human activity patterns, and animal-movement data is a priority for future work to better partition natural aerosolization and deposition from anthropogenic inputs. Our conservative filtering likely underestimates, rather than overestimates, true cross-medium signal, as genuine low-abundance transfers may be obscured.

We also acknowledge that while land-use data can provide valuable contextual information, such data are often coarse, difficult to obtain at appropriate spatial and temporal scales, and do not necessarily map directly onto specific eDNA transport pathways. As such, correlations between surrounding land use and individual exogenous detections should be interpreted cautiously and cannot, on their own, establish causation.

An important caveat is that our passive tray samplers capture deposited material (aerosols and settled particles) rather than filtering a known air volume. Results should therefore be interpreted as deposited airborne eDNA concentrations reflecting integrated deposition over 24 h, not calibrated airborne volumetric concentrations. Active pumping systems that filter defined air volumes would enable direct volumetric comparisons, though at substantially higher equipment and power costs.

By fitting two targeted GLMs, one for water-to-air transfers among aquatic and amphibious taxa, and one for air-to-water transfers among terrestrial taxa, we account for each group’s unique shedding and transport pathways and avoid forcing a single model to fit all eco-hydrological and atmospherical contexts. By linking our study-specific read-count benchmarks to dispersal mechanics, we illustrate how practitioners could, in principle, plan sampling regimes to achieve a desired detection probability, while noting that these thresholds (≈660 water reads, ≈14 air reads) will vary with species traits (e.g., shedding rate, activity, habitat position), sequencing depth, and laboratory protocols, and thus should be calibrated for each new system. By spanning taxa from salmon to amphibians, birds, and mammals, our results fulfill the conceptual “air-water eDNA continuum” framework, demonstrating its applicability across the vertebrate taxa observed here.

These abundance-dependent patterns demonstrate that more abundant taxa are more easily and predictably detected in both media and are more likely to cross the air-water boundary. The specific read-count thresholds (∼660 water reads, ∼14 air reads for 50% cross-medium detection) are specific to our sampling configuration (1 L water grabs, 750 cm^2^ · 24 h passive trays) and should not be generalized without site-specific calibration. Without volumetric air-flow or deposition-flux measurements, air results are best reported as effort-normalized deposition (reads per 750 cm^2^ · 24 h) rather than per-volume concentrations. Future work pairing passive trays with active volumetric samplers and/or standardized deposition collectors would enable true cross-study normalization and predictive modeling of cross-medium transfer dynamics.

### Temporal synchrony and stochastic boundaries

If water and air truly share eDNA, their temporal patterns should echo one another, and indeed, our time-series data aligned well. Peaks in Coho and Chinook salmon eDNA in water were mirrored in passive air traps over the same 24 h sampling window (i.e., air traps were deployed immediately after each water grab), while North American Beaver and Raccoon produced concordant but more gradual rises and falls. For abundant species like salmon, this synchrony confirms that shedding, aerosolization, and deposition operate on comparable timescales across media, enabling airborne eDNA to serve as a faithful, near-real-time sentinel of aquatic population pulses. Occasional anomalies, such as an isolated Raccoon eDNA pulse in air with no matching water signal, likely reflect discrete terrestrial shedding events or weather-driven dispersal, but these do not undermine the overall alignment. By contrast, Wild Turkey eDNA was recovered in both air and water only during the first sampling event on consecutive days, after which it never reappeared in either medium, highlighting how low-abundance terrestrial signals may flicker at the very edge of the continuum. We did not observe turkeys during fieldwork, and this brief, low-read signal likely reflects a transient or anthropogenic input (e.g., feathers or food waste), although Wild Turkeys are known from the region at low abundance, and the detection may simply reflect their rarity. Importantly, the degree of synchrony itself scales with abundance (see Figure S3), confirming that more abundant species exhibit tighter air-water coupling, while rarer taxa remain stochastic.

Despite the synchrony observed for abundant species, this continuum frays at low abundances. While metabarcoding read counts are not perfect measures of absolute biomass, our experimental design’s intentional absence of post-PCR normalization means that raw reads provide an index of relative abundance^26^ that we use to establish operational detection thresholds. Our detection-frequency analysis shows that taxa yielding on the order of 10 reads or fewer appear erratically, with detection rates often below 10%. When targeting rare taxa, the data clearly show that exogenous cross-medium detections become effectively random with respect to the read counts in the source medium. In these cases, it is far more efficient to concentrate sampling effort in the medium where the species is expected, intensively replicating water grabs for aquatic taxa or air traps for terrestrial taxa, rather than chasing sporadic spillover signals. For rare or low-biomass taxa, reliable monitoring requires scaled-up replication, typically three to five times more traps or water grabs, to overcome stochastic detection patterns and achieve desired confidence levels.^16^ Recognizing these stochastic boundaries is essential for conservation applications targeting elusive or endangered species, where effort must be matched to the expected eDNA yield to avoid false negatives.

Beyond these practical thresholds, our data hint at richer dynamics yet to be explored. Quantifying time-lagged cross-correlations between water and air eDNA for different taxa could reveal species-specific transport kinetics—how quickly each organism’s DNA moves through the continuum under varying flow regimes, wind conditions, or diel activity cycles. Such insights would allow adaptive sampling schedules, timed to coincide with peak shedding or optimal transport conditions, further sharpening the power of bidirectional monitoring.

Finally, taxa yielding on the order of 10 reads or fewer exhibited detection frequencies below 10% across samples, providing a practical rule of thumb for our dataset, but one that may vary with sequencing depth, primer choice, and local eDNA concentrations. Strong, abundant eDNA signals produce near-immediate cross-medium echoes, whereas rare signals demand targeted, medium-specific sampling coupled with intensified replication. By mapping these stochastic boundaries, we equip practitioners with the knowledge to deploy continuum-based surveys both efficiently and effectively.

### Frontiers and near-term outlook

Beyond vertebrate eDNA, similar aerosolization and deposition processes have been documented for microplastics,^5^^,^^6^^,^^27^ soil eDNA in rain-wash events,^17^ and airborne microbial DNA recovered over Antarctic deserts.^28^ The empirical framework we establish opens multiple avenues for immediate applications. First, the passive open-water trays require no specialized equipment, just a plastic tray with deionized or mineral water,^11^ making them ideal for remote, resource-limited settings where traditional sampling is impractical. Managers can deploy “passive deposition samplers” in riparian corridors to generate holistic snapshots of vertebrate communities, democratizing biodiversity monitoring by engaging citizen scientists, including hikers, tribal monitors, and school groups, to participate in cross-medium campaigns.

Second, the continuum paradigm underpins powerful early-warning systems.^29^ Paired air-water eDNA deployments could flag the first arrivals of non-native taxa before they are caught by visual surveys or camera traps.^30^ Likewise, restoration projects reintroducing fish or amphibians into degraded waterways could leverage airborne eDNA as a rapid, noninvasive sentinel of colonization success,^31^ detecting large breeding-driven eDNA pulses in both water and air and providing real-time feedback on project outcomes.^32^^,^^33^

Third, the framework extends naturally to ecosystem monitoring and One Health surveillance. Pathogenic microbes and viruses should follow the same cross-medium dynamics: waterborne pathogens aerosolized by bubble bursts or rainfall splashes may become detectable in air, while airborne pathogens settling onto water surfaces could signal emerging contamination events.^34^^,^^35^ Paired sampling thus offers a unified sensor network for zoonotic and waterborne threats at wildlife-human interfaces, enhancing early detection and response capabilities.^36^

Finally, our abundance-driven thresholds and temporal synchrony lay the groundwork for predictive eDNA modeling. By integrating read-count benchmarks with particle-size distribution data, hydrological measurements, aerosol physics, and eDNA decay kinetics,^37^^,^^38^^,^^39^ one can forecast cross-medium hotspots much like air-quality models predict pollutant plumes. Coupled with real-time meteorological and flow sensors,^40^ such predictive tools could power dynamic eDNA dashboards, showing automated alerts and guiding adaptive sampling and triggering rapid management interventions in the face of biodiversity pulses or contamination events.

### Limitations of the study

Several limitations should be considered when interpreting these results. Our passive open-water tray samplers capture deposited material (aerosols, settled particles, and splash) rather than filtering a known air volume, so air results are reported as effort-normalized deposition (reads per 750 cm^2^ · 24 h) and cannot be converted to volumetric airborne concentrations without additional flux measurements. The cross-medium detection thresholds (∼660 water reads, ∼14 air reads for 50% detection probability) are operational values specific to our sampling configuration (MiFish-U 12S marker, MinION R10.4.1, 1 L water grabs, 750 cm^2^ · 24 h passive trays) and should not be generalized across sites, samplers, or taxa without site-specific calibration. Our weekly sampling cadence and occasional gaps limited formal cross-correlation or lag estimation; denser (daily or sub-daily) sampling would be needed to quantify temporal lags in cross-medium eDNA transfer. We also cannot fully distinguish abiotic cross-medium transport (aerosolization, deposition) from biotic vectors (predators, scavengers) or anthropogenic inputs (food waste, pet activity) without experimental manipulation. More broadly, the study was conducted at two urban-wildland interface sites during a single autumn salmon spawning season; the generalizability of our findings to other ecosystems, seasons, and taxa assemblages remains to be tested. Finally, while our intentional absence of post-PCR normalization preserves the relationship between read counts and template abundance, absolute read counts from nanopore sequencing carry higher per-read error rates than short-read platforms, and our operational thresholds should be interpreted accordingly.

## Resource availability

### Lead contact

Further information and requests for resources should be directed to and will be fulfilled by the lead contact, Yin Cheong Aden Ip (adenip@uw.edu).

### Materials availability

This study did not generate new unique reagents. All materials used are commercially available as described in the Key Resources Table.

### Data and code availability

• Raw sequences have been deposited in the NCBI SRA ID: PRJNA1414699. All data needed to evaluate the conclusions in this paper are available in the main text and/or the supplemental information. Data reported in this paper will be shared by the lead contact upon request.
• The bioinformatics pipeline code used in this study is available in Methods S1.
• Any additional information required to reanalyze the data reported in this paper is available from the lead contact upon request. No materials were subject to material transfer agreements (MTAs).

## Acknowledgments

We thank Natasha Kacoroski, Larry Franks, and the dedicated volunteers at Friends of the Issaquah Salmon Hatchery for their generous support in the field. We are also grateful to Travis A. Burnett and Darin Combs at the Washington Department of Fish and Wildlife for facilitating access and allowing field experimental work at the Issaquah Hatchery. We acknowledge funding support from Oceankind and The David and Lucile 10.13039/100000008Packard Foundation [grant no. 2021-72609], and we thank the Center for Environmental Genomics at the University of Washington for providing access to high-performance computing resources. Graphical abstract created with BioRender.com.

## Author contributions

Y.C.A.I. conceived the study. Y.C.A.I. and E.A.A. designed the field and laboratory protocols. Y.C.A.I. and P.F.P.B.-D. jointly designed the downstream bioinformatic analyses. P.F.P.B.-D. conducted the bioinformatic analyses, while Y.C.A.I, G.G., and P.F.P.B.-D. conducted the statistical analyses with inputs from R.P.K. The fieldwork was performed by Y.C.A.I. and E.A.A., while Y.C.A.I. wrote the manuscript. R.P.K. supervised the project, contributed to conceptual guidance, and provided critical revisions. All authors contributed to the study design and approved the final manuscript.

## Declaration of interests

The authors declare no competing interests.

## STAR★Methods

### Key resources table

**Table:** 

REAGENT or RESOURCE	SOURCE	IDENTIFIER
**Chemicals, peptides, and recombinant proteins**		

DNA/RNA Shield	Zymo Research	Cat# R1100-250
DNeasy Blood & Tissue Kit	QIAgen	Cat# 69504
Phusion HF Master Mix	ThermoFisher	Cat# F531L
AMPure XP beads	Beckman Coulter	Cat# A63881
DMSO	Sigma-Aldrich	Cat# D8418
BSA	New England Biolabs	Cat# B9000S
10% bleach (sodium hypochlorite)	Commercial	N/A

**Critical commercial assays**		

SQK-LSK114 Ligation Kit	Oxford Nanopore Technologies	Cat# SQK-LSK114
R10.4.1 MinION Flow Cells	Oxford Nanopore Technologies	Cat# FLO-MIN114
Qubit dsDNA HS Assay Kit	ThermoFisher	Cat# Q32851

**Oligonucleotides**		

MiFish-U-F: 5′-GCCGGTAAAACTCGTGCCAGC-3′	Miya et al.^41^	N/A
MiFish-U-R: 5′-CATAGTGGGGTATCTAATCCCAGTTTG-3′	Miya et al.^41^	N/A
96 custom 14-bp barcode tags	Ip et al.^42^	N/A

**Deposited data**		

Raw Nanopore sequencing data	This paper	NCBI SRA: PRJNA1414699

**Software and algorithms**		

Dorado v0.9.1	Oxford Nanopore Technologies	https://github.com/nanoporetech/dorado
ONTBarcoder v2.3	Srivathsan et al.^43^	https://github.com/asrivathsan/ONTbarcoder
VSEARCH	Rognes et al.^44^	https://github.com/torognes/vsearch
LULU	Frøslev et al.^45^	https://github.com/tobiasgf/lulu
BLASTn v2.15.0+	NCBI	https://blast.ncbi.nlm.nih.gov
TaxonKit	Shen and Ren^46^	https://github.com/shenwei356/taxonkit
Kraken2 v2.1.2	Wood et al.^47^	https://github.com/DerrickWood/kraken2
R v4.4.1	R Core Team	https://www.r-project.org
vegan R package	Oksanen et al.^48^	CRAN
circlize R package	Gu et al.^49^	CRAN
ggplot2 R package	Wickham^50^	CRAN
cowplot R package	Wilke^51^	CRAN
MinKNOW v22.12.5	Oxford Nanopore Technologies	N/A

**Other**		

Smith-Root Citizen Science eDNA Sampler	Smith-Root	https://www.smith-root.com
5.0 um MCE Filter Membranes	Sterlitech	Kent, WA, USA
Polypropylene tray (25 x 30 x 10 cm)	Commercial	N/A
3 L Nalgene Cantene Carboy	Nalgene	N/A
MSI Raider 18 HX (NVIDIA RTX 4090)	MSI	N/A
MinION MK1B device	Oxford Nanopore Technologies	N/A

### Experimental model and study participant details

Omitted as our study does not involve biological models.

### Method details

#### Study sites and sampling design

Between 26 August and 18 November 2024, we conducted 17 overnight sampling trips at two urban–wildland interface sites along Issaquah Creek, Washington, USA. Confluence Park (47.535711° N, –122.039819° W) is a public riparian area with mixed deciduous and coniferous vegetation and intermittent pedestrian traffic. Issaquah Hatchery (47.529501° N, –122.039133° W) is ∼2 km upstream of Confluence Park, and comprises managed salmon-rearing ponds bordered by secondary forest. We scheduled sampling to encompass the October 2024 peak in coho salmon (*Oncorhynchus kisutch*) spawning, based on hatchery visual counts and weekly escapement reports. On each trip, we deployed an open-water tray sampler for passive airborne eDNA at ∼09:00 h and retrieved it ∼24 h later, immediately followed by collection of a matched river-grab water sample. We used paired collection methods throughout: a passive open-water tray (air) that integrates deposited airborne material over ∼24 h (not a volumetric air filter), and a 1 L river-grab (water) filtered through 5.0 μm MCE membranes; below we refer to them as “tray (air)” and “grab (water)” for consistency. Throughout this study, 'medium' refers to the sampling medium (air vs. water), whereas 'habitat affinity’ refers to species' ecological affinity (aquatic, amphibious, or terrestrial).

#### River water sample collection (water)

At the start of each trip, we collected 3 L water samples from the surface of the river using sterile 3 L Nalgene Cantene carboys. Water sampling location was immediately adjacent to air sampler locations. Each 3 L sample was subdivided into three 1 L aliquots, and each aliquot was filtered on site through a 5.0 μm Sterlitech MCE filter (Sterlitech, Kent, WA, USA) via the Smith-Root Citizen Science vacuum pump (Smith-Root, Vancouver, WA, USA). Filter membranes were placed in 1.5 mL DNA/RNA Shield (Zymo Research, USA), transported at room temperature, and then frozen at –20°C within four hours. Of the three 1 L replicates, only one was used for metabarcoding. One field blank (1 L Milli-Q water) was processed at the beginning of each trip through a fresh tubing to check for contamination. Field blanks were preserved and analyzed identically to environmental samples (see laboratory processing and sequencing below for contamination control procedures). All sampling gear was decontaminated with 10% bleach and rinsed with deionized water between uses.

#### Passive open water tray (air)

At each site, a polypropylene tray (25 × 30 × 10 cm; surface area ≈750 cm^2^) was filled with 2 L of molecular-grade deionized water^11^ At Confluence Park, the tray was deployed approximately 5 m inland from the riverbank, positioned approximately 1 m above ground level. At the Issaquah Hatchery, the tray was deployed on the fish ladder railings at the upper level of the site, which is elevated approximately 3 m above the water surface of the creek flowing below. After 24 h deployment, tray water was immediately filtered in the field through a 5.0 μm Sterlitech MCE filter using a Smith-Root Citizen Science vacuum pump (see river-water sample collection above).The resulting filter membranes were then treated identically to those from water samples.

Our open water tray (air) samplers integrate both airborne aerosols and deposited particles over 24 hours, including potential contributions from rain splash, gravitational settling, and bubble-burst aerosolization. We therefore treat the air signal as “deposited air eDNA” with units of deployment effort being hours or days (under the assumption that longer deployment will likely capture more eDNA) rather than a volumetric air concentration. This passive approach requires no electricity and costs <$10 per deployment, making it practical for citizen science and remote monitoring, though it cannot be converted to per-volume air concentrations without additional flux measurements.

Air samplers were deployed for ∼24 h and water samples were collected as 1 L grab samples at ∼09:00 (Table S1 lists exact dates, times, and weather). The passive tray integrates deposited material over a 750 cm^2^ water surface; no air-flow or deposition-flux was measured. Accordingly, we report air results as an effort-normalized deposition unit (reads per 750 cm^2^·24 h) and interpret read counts within-medium, as cross-medium summaries emphasize co-detections rather than absolute read-count contrasts.

#### Laboratory processing and sequencing

Frozen filter membranes from both media (water grabs and air trays) were thawed on ice. For each membrane, 300 μL of DNA/RNA Shield preservative in which the filter was stored was used directly for extraction with the QIAgen DNeasy Blood & Tissue Kit, following manufacturer protocols. We omitted the prior proteinase-K digestion because filters were preserved in DNA/RNA Shield, a chaotropic preservation/lysis buffer that disrupts proteins and inactivates nucleases; given our short (∼170 bp) target amplicon, an additional protease step offered little benefit while adding handling and contamination risk.^42^ Extracts were quantified via Qubit High-Sensitivity assays and stored at –20°C until amplification. We targeted a ∼170 bp fragment of vertebrate mitochondrial 12S using only the MiFish-U primer set,^41^ with the following sequences: MiFish-U-F: 5′-GCCGGTAAAACTCGTGCCAGC-3′; and MiFish-U-R: 5′-CATAGTGGGGTATCTAATCCCAGTTTG-3′.

To enable multiplexing and error-robust sample assignment, 96 custom 14 bp tags were appended to the 5′ end of each primer.^42^ Each 20 μL PCR contained 10 μL Phusion HF Master Mix (ThermoFisher), 0.5 μL DMSO (3% v/v), 0.5 μg μL^-1^ BSA, 2 μL forward primer (5 μM) and 2 μL reverse primer (5 μM; final 0.5 μM each), and 5 μL extract. Thermocycling was: 95°C for 5 min; 35 cycles of 95°C for 30 s, 60°C for 30 s, 72°C for 45 s; and a final extension of 72°C for 5 min. Duplicate reactions per sample were pooled and purified with AMPure XP beads (0.7×). To assess contamination, we included triplicates of no-template control (NTC; molecular-grade water) that were carried through amplification and sequencing alongside environmental samples. PCR and Field blanks (described in Methods 2.2) were processed and analyzed identically to environmental samples. Furthermore, all molecular work was conducted in a 10% bleach and UV-treated PCR-clean hood to minimize contamination.

Libraries were prepared with the Oxford Nanopore SQK-LSK114 ligation kit following standard protocols, then loaded onto R10.4.1 MinION flow cells for 48 h runs on a MK1B device using MinKNOW v22.12.5.

#### Bioinformatics pipeline

Nanopore bioinformatics processing followed pipelines developed in Ip et al. (2026).^42^ Raw POD5 files were basecalled with Dorado v0.9.1 in Super-Accurate (SUP) mode on an MSI Raider 18 HX laptop outfitted with an NVIDIA RTX 4090 GPU. The resulting FASTQ reads were demultiplexed, primer and tag-trimmed using ONTBarcoder2.3^43^ (Data S4) with a minimum read length of 200 bp and allowing up to two mismatches per tag. This step also splits self-ligated concatemer reads, ensuring high-confidence assignment of each sequence to its originating sample.

Following demultiplexing, we performed within-sample dereplication and clustering using VSEARCH.^44^ First, each sample’s FASTQ was converted to FASTA and dereplicated at full length, retaining all unique sequences. These dereplicated reads were then clustered at 98% identity to collapse sequencing noise and generate representative centroids for each operational taxonomic unit (OTU). We next computed a SHA-1 hash for each centroid sequence and assembled an OTU table of raw read counts per sample, facilitating downstream filtering and normalization.

To refine this initial OTU table, we applied the LULU curation algorithm^45^ in R, which identifies and merges artifactual OTUs based on sequence similarity and co-occurrence patterns. Using default settings (minimum match = 84%, minimum relative co-occurrence = 0.95), LULU removed OTUs likely arising from PCR or sequencing errors, yielding a curated table of high-confidence centroids. We then reconstructed a filtered OTU count matrix by retaining only those centroids that passed LULU curation.

Surviving centroids were assigned taxonomy via BLASTn against the NCBI eukaryote “nt” database, as of January 2025. We ran BLASTn (version 2.15.0+) with parameters set for high stringency (≥96% identity, word size = 30, e-value ≤ 1e-40, maximum 50 targets) and output formatted to capture scientific and common names, alignment metrics, and taxonomic identifiers. This BLAST output provided a pool of putative taxonomic hits for each centroid.

To derive the most likely taxonomic identity and rank for each sequence, we employed TaxonKit^46^ to compute the lowest common ancestor (LCA) —the most specific taxonomic rank shared by all top-identity BLAST hit per hash. The resulting LCA table was merged back with BLAST metadata to produce a final annotated OTU dataset. To avoid confounding ecological interpretation with anthropogenic “food” signals, we prespecified conservative exclusion criteria and applied them uniformly. A taxon was excluded only when all three conditions were met: (i) it belonged to domestic livestock or poultry lineages that do not occur naturally at our sites; (ii) its detections were low-copy and lacked corroboration from site lists or regional occurrence records; and (iii) the same taxon was present in field blanks and/or PCR no-template controls at comparable or higher relative abundance. Taxa meeting all three conditions were omitted from community summaries, while all raw assignments—including excluded taxa—are retained in Data S3 and S4 for transparency. Lastly, we manually curated taxonomic assignments. Each LCA-annotated OTU was manually evaluated for ecological plausibility within the Issaquah Creek watershed. We collapsed several ambiguous records to genus level (e.g., *Cottus* sp., *Microtus* sp.) where species-level resolution exceeded the marker’s discriminatory power or lacked local reference sequences. Singleton OTUs (those with only one read across all samples) were removed as probable artefacts. A small number of salmonid sequences that persisted despite these filters, and that only appeared alongside high-abundance salmon in the same samples, were deemed residual sequencing errors and manually excised. Rare salmonid names excluded at this step were within-genus congeners (e.g., *Oncorhynchus nerka*, *O*. *keta*) that appeared only alongside high-abundance Coho/Chinook salmon and lacked independent corroboration. Given MiFish-U 12S’s limited within-genus resolution, we collapsed these to *Oncorhynchus* sp. (removing singletons as artefacts). The final dataset comprised 39 vertebrate taxa confidently detected across 27 environmental DNA samples spanning both media (14 air-tray and 13 river-grab samples).

Reads arising from PCR no-template controls (NTCs) were not clustered, but instead were classified with Kraken2 (v2.1.2) against the NCBI core_nt database at 0.05 confidence to screen for broad contamination of vertebrate and salmonid signals,^42^ whereas field blanks were classified using the same bioinformatics pipeline as environmental samples. All negative control results are summarized in Table S2.

### Quantification and statistical analyses

All statistical analyses were carried out in R version 4.4.1. We began by assessing taxonomic overlap between waterborne and airborne eDNA: raw read counts for each species were converted to presence–absence, and the numbers of taxa unique to each medium or shared across both were depicted in a chord diagram (*circlize*;),^49^ with ribbon thickness proportional to log_10_(reads + 1) and wedges colored by habitat affinity (aquatic, amphibious, terrestrial).

Since our MinION workflow did not include any pre-sequencing normalization of library concentrations as is common in Illumina workflows. We treat total ONT read counts (per sample) as an index of abundance of the underlying amplifiable signal in the sample (unlike in Illumina datasets, where normalization removes this relationship). To test whether the distribution of taxa differed more than expected by chance across habitat groups and media, we again converted all read counts into presence–absence. Next, we grouped species by habitat affinity (aquatic, amphibious, terrestrial) and tabulated, for each habitat, how many species were detected in air versus in water. This yielded a 3 × 2 contingency table (rows: Aquatic, Amphibious, Terrestrial; columns: “Detected in Air” vs. “Detected in Water”). We then applied Pearson’s Chi-square test to evaluate whether habitat affinity was associated with differences in detection frequency between air and water, with significance assessed at p < 0.05.

Next, for exogenous DNA transfer, we quantified the ease with which DNA “spills over” from one medium to the other by fitting binomial generalized linear models (i.e., logistic regressions) – that is, we are interested in the probability of detection in one medium (air or water) as a function of its abundance in the other medium. For each taxon *i* detected in sample *j* in medium *m* where *m* ∈{*w, a*} (*w* for water and *a* for air), we modeled the probability of its detection *p* as a function of the log_10_ +1 reads (R) in the opposite medium *k*, where *k* ∈{*w, a*} ∖{*m*} (i.e., if *m=w* then *k=a* for air and vice-versa) using a logistic regression with intercept *β*0 and slope *β*1, as

$$p_{ijm}=logit^{-1}\left({\beta 0}_{m}+{\beta 1}_{m}{\times R}_{ijk}\right)$$

Models were fitted in R using glm (Y ∼ R, family = “binomial”), where y is the 0 or 1 response variable. The resulting logistic curves illustrate how increasing abundance in the source medium predicts cross-medium detections, again under the assumption that greater numbers of reads reflect greater abundance of underlying template molecules in the context of unnormalized ONT sequencing. Since water sampling filtered 1 L per sample while air sampling collected whatever settled onto a 750 cm^2^ tray over 24 h, our logistic-regression curves reflect two very different sampling substrates. Ideally, we would convert air-trap deposition into an equivalent air volume to standardize “reads per unit effort.” In the absence of that normalization, cross-medium detection thresholds should be interpreted qualitatively rather than as absolute concentration comparisons.

To examine temporal concordance between media, we calculated a relative-read abundance index (“eDNA-index”)^16^ for each taxon in each sample: species read counts were divided by the sample’s total reads to yield proportions, then normalized by each taxon’s maximum proportion (across all samples) to scale the index from 0 to 1. We restricted analyses to taxa that (i) were detected in both media on multiple sampling dates (to avoid single co-detections), and (ii) had sufficient non-zero reads to compute stable eDNA-index trajectories. From this subset, we selected six focal species representing aquatic, amphibious, and terrestrial habitat affinities (Coho Salmon, Chinook Salmon, Rainbow Trout, North American Beaver, Raccoon, Wild Turkey). We plotted a time-series of this index for these six species, visualizing whether peaks and troughs align across air and water, and quantified air–water temporal concordance by pairing each taxon’s eDNA-index time series for air and water and treating non-detections as zeros. For each species we fit a lag-0 ordinary least-squares model of air index on water index and used R^2^ as a descriptive measure of concordance. Given the weekly cadence and occasional gaps (Table S1), formal cross-correlation is underpowered for precise lag estimation, so we report lag-0 R^2^.

We further explored how detection reliability varies with sampling effort by computing, for each species and medium, the proportion of samples in which it was detected (“detection frequency”) and plotting those frequencies against the square root of total reads per species. This analysis illustrates the diminishing returns of additional sampling for common taxa versus the high effort required to capture rare, stochastic signals.

Finally, to characterize community-level patterns, we generated both Jaccard (presence–absence) and Bray–Curtis (on log10-transformed read counts) dissimilarity matrices and performed non-metric multidimensional scaling in *vegan*^48^ to visualize sample clustering by medium and site. We then used PERMANOVA (adonis2, 999 permutations, by = ‘margin’) to partition the variance in community composition using the model: dissimilarity ∼ Medium + Location + Week + Day. This model tests the marginal effect of each predictor (sampling medium, site location, sampling week, and sampling day) on community composition while controlling for the other terms.

All visualizations were produced with *ggplot2* and assembled into multi-panel figures using *cowplot*.

## Supplemental information

Document S1. Figures S1–S3 and Tables S1 and S2

Methods S1. Bioinformatics pipeline code for post-demultiplexing sequence processing

Data S2. Complete read count matrix for all samples and field negative controls

Data S3. Complete Kraken2 classification output for PCR negatives

Data S4. Sample demultiplex sheet
