# Supplementary material for: Vertebrate biodiversity via eDNA at the air-water interface
Source: iScience. 2026 Apr 9;29(5):115682. doi: 10.1016/j.isci.2026.115682 (PMC13141742; doi:10.1016/j.isci.2026.115682)
Supplement: Document S1. Figures S1–S3 and Tables S1 and S2 [file mmc1.pdf]

iScience, Volume *29*

Supplemental information

Vertebrate biodiversity via eDNA

at the air-water interface

Yin Cheong Aden Ip, Pedro F.P. Brandão-Dias, Gledis Guri, Elizabeth Andruszkiewicz Allan, and Ryan P. Kelly

1. Supplementary Figures and Table

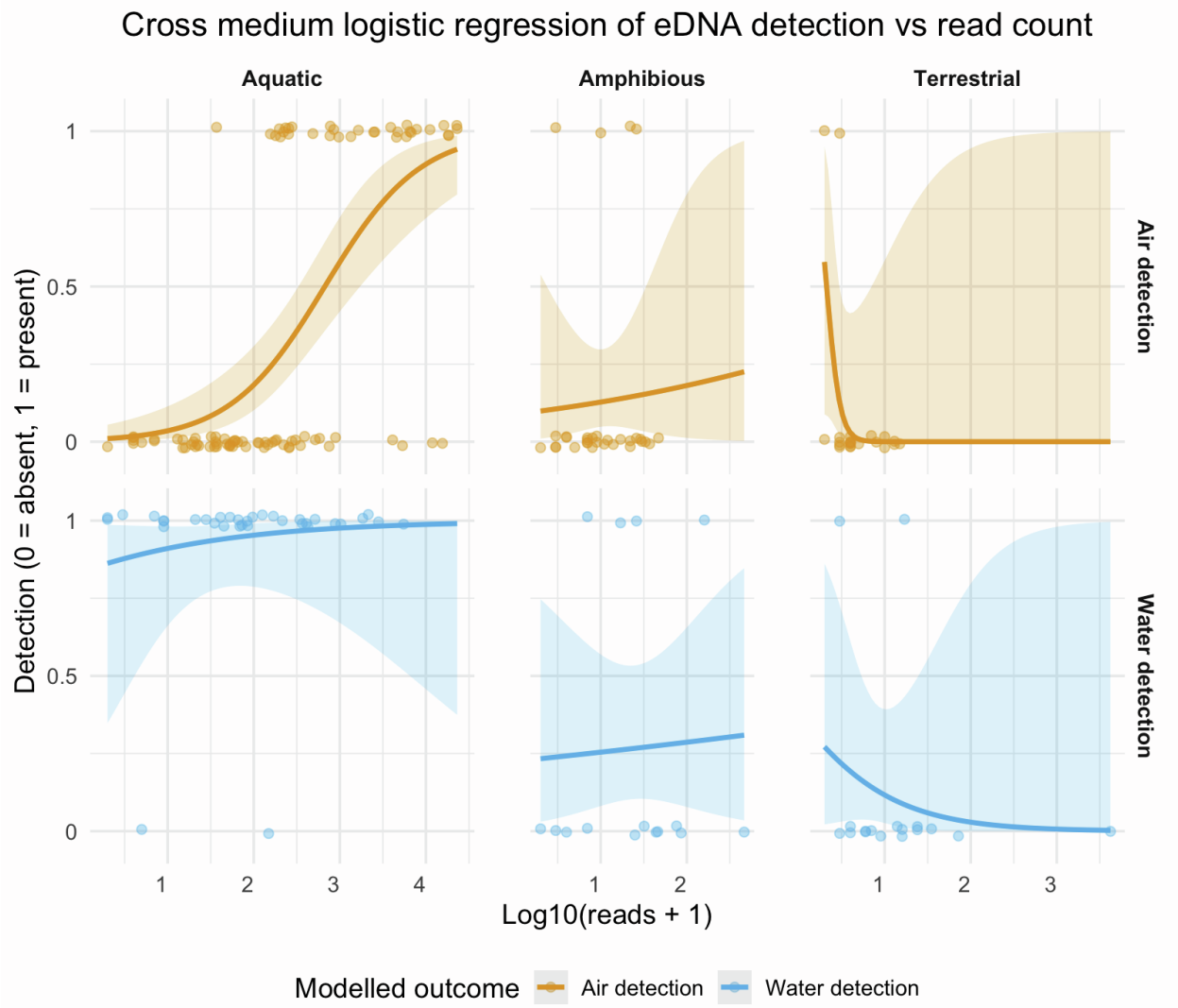

Supplementary Figure S1. Cross-medium logistic regression of eDNA detection probability by ecological guild and medium. Each column shows the subset of species living in one habitat guild: Aquatic, Amphibious, and Terrestrial. Each row shows detection in the alternate medium: Air detection (top row) and Water detection (bottom row). On the x-axis is the $\log_{10}$-transformed read count (reads + 1) in the source medium; on the y-axis is the binary detection outcome in the target medium (1 = present, 0 = absent). Gold points and curves correspond to the probability of detecting a taxon (of each specific guild) in air as a function of its waterborne eDNA abundance; blue points and curves correspond to the probability of detecting a taxon (of each specific guild) in water as a function of its airborne eDNA abundance. Points are jittered vertically for clarity, and the smooth lines are fitted binomial GLMs. Shaded ribbons around each curve represent the 95% confidence intervals of the logistic-regression fit.

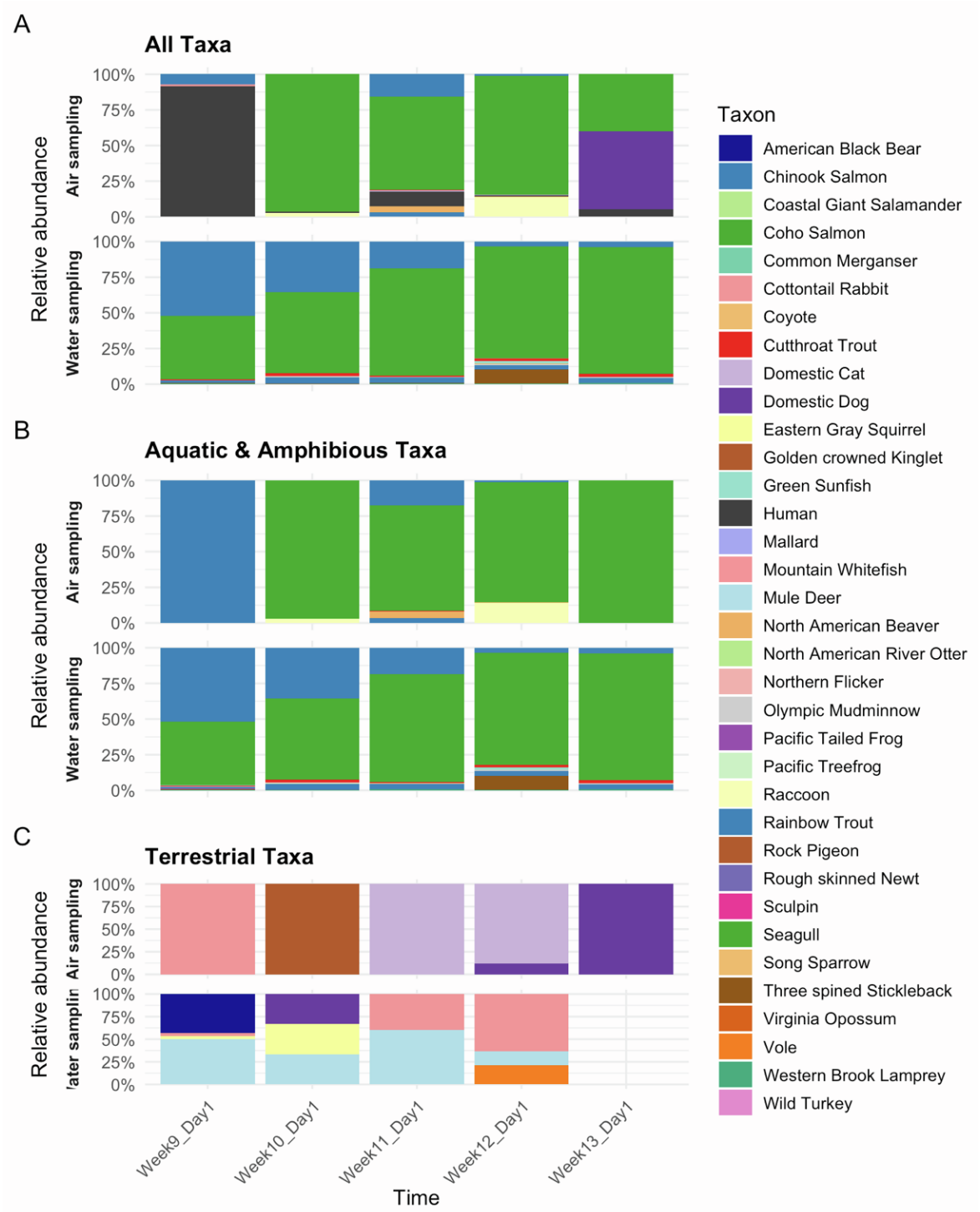

Supplementary Figure S2. Relative abundance of eDNA-detected taxa at Confluence Park over time, comparing air versus water sampling. (A) All detected taxa. (B) Only aquatic and amphibious taxa. (C) Only terrestrial taxa. In each panel, the top row shows air-sampling filter results and the bottom row shows water-sampling filter results, with stacked bars representing the proportional (100%) taxon composition over five weeks. Taxa are keyed by color in the legend to the right.

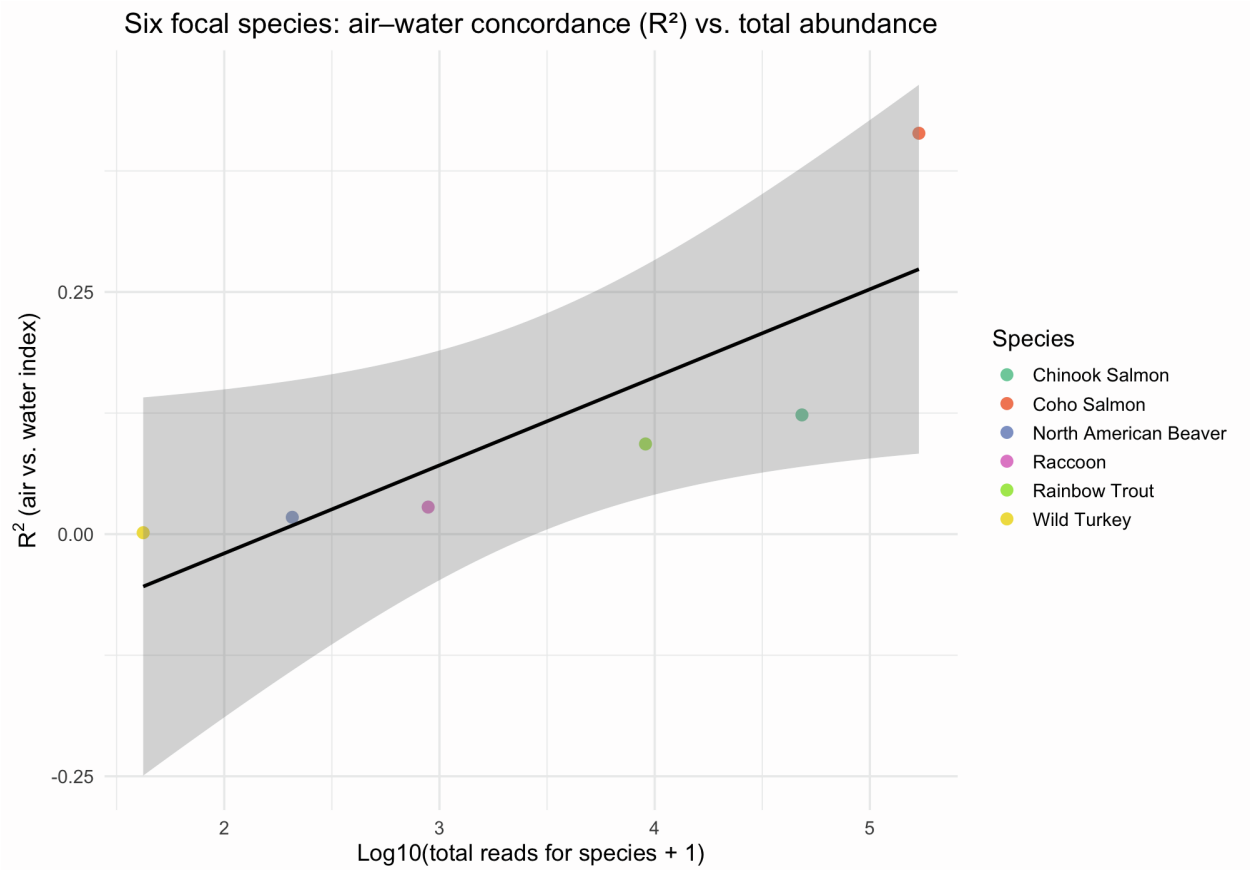

Supplementary Figure S3. Species-level air–water concordance (R^2) as a function of overall eDNA read abundance. Each point represents one of the six focal taxa (colored by species), plotting its R^2 from a linear regression of air-index versus water-index against $\log_{10}$(total reads + 1) across all samples. The solid black line is the best-fit linear model ($\beta \approx 0.10 \pm 0.03$ SE, $p = 0.02$, adj-$R^2 = 0.48$), with the shaded ribbon showing the 95% confidence interval.

Supplementary Table S1. Sampling metadata and sequencing yield for paired airborne and waterborne eDNA samples analyzed in this study. Air samples were deployed at approximately 09:00 and retrieved 24 hours later. Water samples were collected at approximately 09:00 on each sampling trip. Rain and precipitation data are from local weather stations for the collection date. PreFilterReads refers to the raw sequencing output while PostFilterReads are the reads retained after quality filtering and bioinformatic processing (see Methods Section 2.5).

Sample	Location	Medium	Week	Day	Date	Rain	Precipitation_mm	PreFilter Reads	PostFilter Reads
Air018	Issaquah Hatchery	Air	Week 1	Day 1	2024 0826	No	0.0	22774	12764
Air024	Issaquah Hatchery	Air	Week 1	Day 2	2024 0828	Yes	0.04	215	97
SAL042	Issaquah Hatchery	Water	Week 1	Day 2	2024 0828	Yes	0.04	23532	7672

Air025	Issaquah Hatchery	Air	Week 1	Day 3	2024 0830	No	0.0	77	48
SAL049	Issaquah Hatchery	Water	Week 1	Day 3	2024 0830	No	0.0	58793	17129
Air036	Issaquah Hatchery	Air	Week 8	Day 1	2024 1017	Yes	0.05	8481	4865
SAL093	Issaquah Hatchery	Water	Week 8	Day 1	2024 1017	Yes	0.05	117360	32543
SAL149	Confluence Park	Water	Week 8	Day 1	2024 1019	Yes	0.16	39648	8787
Air042	Confluence Park	Air	Week 9	Day 1	2024 1022	Yes	0.2	1749	1208
SAL101	Confluence Park	Water	Week 9	Day 1	2024 1023	Yes	0.01	141255	35081
Air048	Issaquah Hatchery	Air	Week 9	Day 2	2024 1024	Yes	0.01	10543	3361
SAL105	Issaquah Hatchery	Water	Week 9	Day 2	2024 1025	No	0.0	67800	20293
Air054	Confluence Park	Air	Week 10	Day 1	2024 1028	Yes	0.62	26740	5710
SAL113	Confluence Park	Water	Week 10	Day 1	2024 1028	Yes	0.62	51333	11526
Air060	Issaquah Hatchery	Air	Week 10	Day 2	2024 1031	Yes	0.32	4464	1975
SAL117	Issaquah Hatchery	Water	Week 10	Day 2	2024 1031	Yes	0.32	28452	7184
Air066	Confluence Park	Air	Week 11	Day 1	2024 1105	Yes	0.19	1530	620
SAL125	Confluence Park	Water	Week 11	Day 1	2024 1105	Yes	0.19	129170	24410
Air072	Issaquah Hatchery	Air	Week 11	Day 2	2024 1107	No	0.0	9770	3171
SAL129	Issaquah Hatchery	Water	Week 11	Day 2	2024 1107	No	0.0	22410	5289
Air078	Confluence Park	Air	Week 12	Day 1	2024 1112	Yes	0.26	16636	3387
SAL137	Confluence Park	Water	Week 12	Day 1	2024 1112	Yes	0.26	30601	7546
Air084	Issaquah Hatchery	Air	Week 12	Day 2	2024 1114	Yes	0.29	4571	1236
SAL141	Issaquah Hatchery	Water	Week 12	Day 2	2024 1114	Yes	0.29	53746	12248

Air090	Confluence Park	Air	Week 13	Day 1	2024 1119	Yes	0.69	152	130
Air096	Issaquah Hatchery	Air	Week 13	Day 2	2024 1121	No	0.0	3179	1080
SAL153	Issaquah Hatchery	Water	Week 13	Day 2	2024 1121	No	0.0	120680	24892

Supplementary Table S2. Summary of detections in negative controls and field blanks. Values indicate total read counts per taxon after bioinformatic filtering. No PCR negative contained salmonid reads. Field blanks show trace vertebrate DNA consistent with ambient aerosols. Field negatives are classified the same way as samples while PCR negatives are classified with Kraken2 core_nt database.

Control type	Sample ID	Total Reads	Representative vertebrates (>1 read)	Salmonid Reads
PCR NTC	NTC_a	1,723	*Esox lucius*	-
PCR NTC	NTC_b	406	*Pempheris schwenkii*, *Canis lupus*	-
PCR NTC	NTC_c	1,851	*Neofelis diardi*	-
Field Blank	SAL041	57	*Lampetra richardsoni*, *Prosopium williamsoni*	*O. tshawytscha* (26)
Field Blank	SAL100	62	*Lampetra richardsoni*	*O. kisutch* (55)
Field Blank	SAL136	335	*Lampetra richardsoni*	*O. kisutch* (113)
Field Blank	Others (n = 8)	11	*Anas platyrhynchos*, *Microtus sp.*	-
